# Standardized observation of neighbourhood disorder: does it work in Canada?

**DOI:** 10.1186/1476-072X-9-6

**Published:** 2010-02-10

**Authors:** Janet A Parsons, Gita Singh, Allison N Scott, Rosane Nisenbaum, Priya Balasubramaniam, Amina Jabbar, Qamar Zaidi, Amanda Sheppard, Jason Ramsay, Patricia O'Campo, James Dunn

**Affiliations:** 1 Applied Health Research Centre, Li Ka Shing Knowledge Institute, St. Michael's Hospital, Toronto, Canada; 2 Department of Physical Therapy, University of Toronto, Toronto, Canada; 3 Department of Obstetrics & Gynecology, Markham-Stouffville Hospital, Toronto, Canada; 4 Centre for Research on Inner City Health, Li Ka Shing Knowledge Institute, St. Michael's Hospital, Toronto, Canada; 5 Dalla Lana School of Public Health, University of Toronto, Toronto, Canada; 6 Institute of Medical Science, University of Toronto, Toronto, Canada; 7 Cancer Care Ontario, Toronto, Canada; 8 FutureHealth Inc., Toronto, Canada; 9 Neurorehabilitation Program, Toronto Rehabilitation Institute, Toronto, Canada

## Abstract

**Background:**

There is a growing body of evidence that where you live is important to your health. Despite numerous previous studies investigating the relationship between neighbourhood deprivation (and structure) and residents' health, the precise nature of this relationship remains unclear. Relatively few investigations have relied on direct observation of neighbourhoods, while those that have were developed primarily in US settings. Evaluation of the transferability of such tools to other contexts is an important first step before applying such instruments to the investigation of health and well-being. This study evaluated the performance of a systematic social observational (SSO) tool (adapted from previous studies of American and British neighbourhoods) in a Canadian urban context.

**Methods:**

This was a mixed-methods study. Quantitative SSO ratings and qualitative descriptions of 176 block faces were obtained in six Toronto neighbourhoods (4 low-income, and 2 middle/high-income) by trained raters. Exploratory factor analysis was conducted with the quantitative SSO ratings. Content analysis consisted of independent coding of qualitative data by three members of the research team to yield common themes and categories.

**Results:**

Factor analysis identified three factors (physical decay/disorder, social accessibility, recreational opportunities), but only 'physical decay/disorder' reflected previous findings in the literature. Qualitative results (based on raters' fieldwork experiences) revealed the tool's shortcomings in capturing important features of the neighbourhoods under study, and informed interpretation of the quantitative findings.

**Conclusions:**

This study tested the performance of an SSO tool in a Canadian context, which is an important initial step before applying it to the study of health and disease. The tool demonstrated important shortcomings when applied to six diverse Toronto neighbourhoods. The study's analyses challenge previously held assumptions (e.g. social 'disorder') regarding neighbourhood social and built environments. For example, neighbourhood 'order' has traditionally been assumed to be synonymous with a certain degree of homogeneity, however the neighbourhoods under study were characterized by high degrees of heterogeneity and low levels of disorder. Heterogeneity was seen as an appealing feature of a block face. Employing qualitative techniques with SSO represents a unique contribution, enhancing both our understanding of the quantitative ratings obtained and of neighbourhood characteristics that are not currently captured by such instruments.

## Background

There is a growing body of evidence that where you live is important to your health [1-4]. Environmental factors (e.g. air quality, proximity to industrial pollutants), neighbourhood income, and neighbourhood structure have all been linked to a variety of health outcomes [5-7]. However the nature of the relationship between neighbourhood conditions and residents' health (and the mediator and moderator factors at play) remains unclear.

Characterizing neighbourhoods and the specific features of neighbourhoods that may contribute to residents' health and well being is a complex and difficult undertaking. Considerable attention has been devoted to the role of neighbourhood poverty (and its potential health effects), in part secondary to trends in recent decades where poverty has become more spatially concentrated in inner-city neighbourhoods, at least in the United States [6,8]. Investigators have linked neighbourhood income to individual-level health outcomes, using either administrative datasets or individual-level survey responses [1,7]. Most studies using objective data sources have relied on census data, which provide information about socioeconomic position of a given census tract relative to others (e.g. median household income), population stability, and ethnic composition of a neighourhood [9]. There are a number of problems with using such census data, one of which is that they provide neither information regarding the social life of the neighbourhood nor the physical characteristics of the built environment [3,9].

Relatively few studies have employed direct observation of neighbourhood environments. Of those that have, most have concentrated on both physical (geographical) features of urban environments and/or on social features to which residents are exposed [10]. While social environmental influences on health have been documented [1,3,4,11], the relationship between neighbourhood disadvantage, physical disorder, and social disorder/social cohesion remains unclear. Much of this research is grounded in the field of criminology [12-14] and has then been adopted as a starting point for health research [15,16]. One influential model (Skogan, 1990)[17] suggests that the more prevalent physical incivilities/disorder become, residents' perceptions of their neighbourhood shift, leading to decreased social cohesion (and increasing crime) ([17] cited in [12]; [14]). Markowitz and colleagues (2001) posit a feedback loop whereby the effect of disorder on neighbourhood cohesion is mediated by fear [12]. The concept of 'social capital' is another theoretical construct underlying much of the neighbourhoods-and-crime literature, and is defined by Sampson and colleagues (1997) as 'collective efficacy' (premised on "'mutual trust and a shared willingness to intervene for the common good' of the community") (Sampson et al., 1997 in Franzini et al., 2005)[18](p. 1136). Such collective efficacy is posited to be comprised of two processes: "social cohesion (the sense of connectedness) and informal social control (the willingness to intervene in community problems)" [18]. However the link between social disorder and social cohesion (and how this relates to health) has yet to be definitively demonstrated. Nevertheless it is important to understand the theoretical assumptions underlying much of the literature on systematic social observations (SSO).

What then does 'neighbourhood disorder' look like? Prior research in the US and the UK has resulted in the development of standardized observational tools in an attempt to quantify features indicative of disorder in both the immediate social and physical environments. These standardized instruments have further informed the development of measurement scales with which to compare neighbourhoods. To date, most studies employing these measures have focused on observational data at the level of randomly selected block faces within specific census tracts [7,14]. Such observational tools are essentially checklists used to inventory and rate aspects of the social and physical environments of each block face. Data are typically aggregated up to whatever definition of neighbourhood is being used, and associations with various health outcomes investigated.

To date, these scales have never been applied in a Canadian context to a range of urban block faces within a single city. While Kohen and colleagues (2002) applied a scale ostensibly capturing both physical and social disorder in a large Canadian sample (n = 3,350), the authors only looked at a very limited range of physical (traffic volume, presence of garbage, building conditions) and social attributes (loitering, hostile behaviour, drunkenness/intoxication) [19]. The focus was at the level of individual families (not at the city or neighbourhood level) and only a very limited portion of the block face surrounding the participants' home (same side of street) was examined. It did not investigate neighbourhoods or their characteristics in depth. A study by Coen and Ross (2006) has applied such scales specifically to the study of Montreal neighbourhood parks, but did not look beyond these particular features [20].

There are several reasons for questioning the applicability and appropriateness of such instruments for studying public health in urban contexts outside the United States. There has been relatively little critical scrutiny of the theoretical assumptions linking physical and social disorder with crime, and their subsequent extension to the arena of public health. Few have questioned the appropriateness of this extrapolation. For example, some investigators drawing on these assumptions have hypothesized that evidence of territoriality and defensible space could represent physical manifestations of social cohesion, suggesting that if a criminal offender were to cross territorial boundaries that residents would take defensive action [14,21]. This appears to us a potentially problematic assumption, and that gardens, shrubs and low railings may not be evidence of such defensive thinking, but may have quite different meanings for residents. Caughy et al. (2001) problematize the assumption that the presence of physical incivilities (presence of trash or graffiti) reflects an 'uncaring' attitude by residents [22], pointing out these may be the combined result of municipal priority setting and resident will [21]. Furthermore, research by Oreopoulos (2005) highlights some important differences between the five largest Canadian cities and their five largest counterparts in the US [23]. Oreopoulos notes that low-income census tracts in Canada are not characterized by segregation of visible minorities (as they are in the US), and Canadian residents of low-income census tracts are exposed to much lower rates of crime than their US counterparts [23]. He indicates that the poverty lines in the two nations are not directly comparable, and that it should not be assumed that the experiences of low-income households in the lowest income census tracts are similar in both countries [23]. Thus it may be that applying such observational tools in urban settings outside the US is of questionable utility, given the stark differences in the social geography of US cities when compared to those of other nations.

In this paper we applied observational tools measuring physical and social disorder (developed for use in US and UK cities) to a specific Canadian urban context. This study is one in a series of investigations regarding neighbourhood-level influences on health being conducted in Toronto, Canada's largest city. Prior to examining linkages with residents' health, it was first necessary to determine if previously-developed observational tools were transferable to the Toronto context. Drawing on prior work by Raudenbush & Sampson (1999), Caughy and colleagues (2001) [18], Weich et al. (2001) and others, [7,9,18,21,24,25] we began from an assumption that "neighbourhood impoverishment" is a "source of neighbourhood social and physical characteristics"[18]. Raudenbush & Sampson (1999) developed a reliable coding method for neighbourhood block faces (using videotaped data), premised on the notion that structural characteristics can influence neighbourhood social organization and collective efficacy [8]. Weich et al. (2001) validated a survey checklist of built environment features for use in UK cities (specifically, structural and building features; use of green, public and vacant spaces; security/safety; and accessibility to amenities). The items chosen for inclusion in our study incorporated those which had previously demonstrated reliability and validity, and whether they made conceptual sense (based on knowledge of the existing literature).

We did not attempt to link the observational data to health outcomes per se in our study, but rather were concerned with testing the elements of these various instruments to determine if they were sensitive to detecting physical and social disorder in a Canadian context. Such validation studies are a necessary first step in this area of research and are of interest to health researchers, aiding them in the interpretation of findings acquired using these instruments. While we originally intended to apply quantitative methods in isolation, it became apparent at the outset of data collection that qualitative techniques would provide important insights regarding the tool's performance. As the research team comprised members with expertise in both methodological approaches, a qualitative component of the study was incorporated into the study design. While this has not been done in prior SSO studies, the rationale for including qualitative approaches is sound. Anthropologists have long recognized the subjective nature of observation (even when undertaken systematically) and we drew upon this rich tradition in the social sciences to inform our study design [26]. It is increasingly recognized that innovative mixed-method approaches are important, because our understanding of quantitative evidence (the measured relationship between variables) can be enhanced by qualitative evidence to understand the 'how' and 'why' of (and processes underlying) those relationships [27]. Cuthchin (2007) and others emphasize the importance of recognizing culture and its implications for geographic spaces/built environments, and their meanings to observers [5,28,29]. To our knowledge the inclusion of qualitative methods represents a unique contribution to the field of SSO.

## Methods

### Data collection procedures

This mixed-methods study entailed compiling an observational tool comprised of the union set of items from four widely used instruments (described below) and testing its performance in a variety of Toronto neighbourhoods. A consensus process with community partners defined the following Toronto neighbourhoods to be surveyed: Eglinton East, South Parkdale, St. Jamestown, and Weston (all considered low-income) and Banbury-Don Mills and North Riverdale (both middle/high income). All four low-income neighbourhoods are characterized by higher incidence of low-income households (e.g. St. Jamestown 47.9%, South Parkdale 46.4%), compared with the mid/high-income neighbourhoods (Banbury-Don Mills 14.3%, North Riverdale 15.6%) (City of Toronto statistics) [30]. The neighbourhoods were defined by existing City of Toronto criteria (which are numerous) and are further defined by Statistics Canada census tracts (for purposes of statistical reporting) [30]. No Toronto neighbourhood is comprised of a single census tract, and each has a minimum population of between 7,000 and 10,000 people. Of the six study neighbourhoods, five had populations > 16,000 (two with populations over 22,000), and one (North Riverdale) had approximately 11,000 persons[30]. Toronto is one of the most ethnically diverse cities in the world and the most diverse nationally; most of its neighbourhoods have significant proportions of new immigrant and visible minority populations. That being said, the four low-income neighbourhoods are considered some of the most ethnically diverse with some of the largest populations of new immigrants in the city [30].

Data collection took place in the six neighbourhoods, in order to determine whether the composite SSO instrument could detect differences between neighbourhoods of disparate median incomes. These neighbourhoods are also characterized by diverse built forms (i.e. characterized by a mixture of residential and commercial structures, varied residential layouts (grid versus curvilinear streets), mixture of high-rise and low-rise buildings, varied population densities, road zoning, etc.).

**Figure 1:**
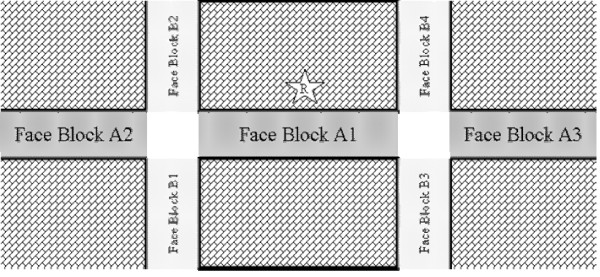
**Block face diagram**.

A block face (BF) was defined as "any street between two intersections" and included both sides of the street (Fig. 1); an intersection was characterized by the presence of street signs, breaks in sidewalks, or a dead end. Due to budget and time constraints, the study was designed to collect data on a maximum of 180 BFs. It was decided that to better allocate resources, the 180 BFs would be distributed in a ratio of 2:1 for low to middle/high income neighbourhoods (120:60). Within each income level, we selected BFs with probability proportional to total number of BFs (497 and 455 respectively for low to middle/high income neighbourhoods). Within each neighbourhood, BFs were listed in a quasi-geographic order, by looking at the neighbourhood street map and recording the streets between two intersections. Systematic random sampling with probability proportional to neighbourhood size, with the appropriate sampling rates, was applied. This meant that for the low-income neighbourhoods, the sampling rate was 120/497 (or 24.15%), and for middle/high income neighbourhoods was 60/455 (or 13.19%). The final sample included 176 BFs.

Data collection occurred at two different times of day (daylight and evening hours) to account for temporal differences in certain variable items (described below). We also conducted repeated observations on a sub-sample of BFs (n = 16 or 9.1% of total BFs) to ensure inter-rater reliability. The answer to each of 56 SSO fixed items was compared between two 'gold standard' trainers and raters, and concordance was indicated by a check in a paper grid. The total number of concordant answers was calculated for each BF, and the proportion of correct answers (or percent agreement) was calculated by dividing it by 56. We further documented the data collection processes using qualitative methods, encouraging raters to reflect on their fieldwork experiences and the perceived utility of the tool in situ. In this report, we examine the properties of the data collected using this mixed-method design; qualitative techniques, descriptive statistics and exploratory factor analysis are employed. The qualitative findings inform the results of the quantitative analysis.

### SSO tool

We reviewed the literature to identify constructs, scales, or items related to SSO of neighbourhoods previously employed in cities in the US and UK [7,21,25]. We created a pilot observational tool that included 98 items from four sources: (1) Raudenbush & Sampson (1999) who reported on items related to the constructs Social Disorder, Physical Disorder and Physical Decay [7]; (2) Caughy et al. (2001) who examined items related to the constructs Physical Incivilities, Territoriality and Available Play Resources [21]; (3) Weich et al. (2001) who investigated the association of individual 'Built Environment' items with depression [25]; and (4) Sastry & Pebley (2003) who employed a public-access neighbourhood survey instrument [24]. The domains of the resulting composite tool included: (1) Characteristics of streets and sidewalks, (2) Residential land use, (3) Non-residential land use (commercial, industrial or vacant), (4) Parks and playgrounds, (5) Commercial establishments, services, and institutions, (6) People, and (7) Incivilities (copies of the pilot SSO instrument are available upon request from the authors).

All observations were completed between August and October 2006. The 98-item checklist was completed by raters between the hours of 09:00 and 15:30. Because some of the SSO items could potentially vary with time of day (e.g. presence of police officer on the BF, number of visible children) a subset of 41 'variable' items was identified. Raters returned to each BF to evaluate these variable items between 17:00 and 20:00 hrs. We consolidated the repeated information of variable items by indicating presence (or attribute) observed either during the first or second observation. In the case of ordinal items, we selected the highest category observed (e.g., *some *garbage was selected over *little *garbage).

All raters underwent a week-long intensive training program conducted by two project coordinators experienced with SSO. Training consisted of in-depth item review via a standardized protocol, training slideshow presentation, and informal classroom exercises. In addition, formal field exercises were conducted in two neighbourhoods which simulated the experience of performing SSOs (similar to the methodology described by Caughy) [21]. Competency of raters was determined by the achievement of a minimum score on the 98-item comprehensive checklist. Per cent agreement was evaluated for 16 BFs (or 9.1% of the total sample of BFs) and confirmed a high level of agreement between raters and the most experienced trainer (median agreement = 89.3%, SD ± 15.6%).

### Qualitative Methods

For the qualitative component of the study, an emergent design was adopted based on the initial implementation of the SSO tool in the neighbourhoods. Raters commented at team meetings (early on in the data collection process) that the tool failed to capture certain aspects of the BFs being evaluated - for example, interactions with residents, raters' sense of personal safety, and aesthetic appeal of the block face. They also voiced concerns that a failure to capture these aspects (owing to the fixed response categories of the observational tool) might result in misrepresentation of the BFs under study. Following group discussions a decision was made early on to create a separate "Comments" section within the SSO tool. This encouraged the observers to document their experiences and additional observations not otherwise effectively captured by the instrument.

All hand-written field comments were transcribed into an electronic text document and labelled with BF and neighbourhood identifiers. The comments were then independently coded by three members of the research team using a content analysis approach. Emergent categories and themes were identified and presented to the qualitative sub-committee for further analysis and discussion. Based on the emergent themes and categories, sub-categories were created.

In addition to the written comments analysis, a focus group discussion was conducted with all raters once data collection had been completed, in order to allow for further elaboration on their field-work experiences. In addition, one of the expert trainers and one of the senior research team members attended the focus group. The focus group (facilitated by a member of the research team with expertise in qualitative research methods - JAP) was audio-taped and transcribed verbatim, providing an additional source of qualitative data for analysis [31]. Topics for discussion included: perceived positive features of the instrument, perceived shortcomings, suggestions for improving the instrument, reflections on fieldwork experiences (including surprises, expectations, the role of social interactions with residents), and perceptions regarding what constitutes an 'appealing' BF. These data served to clarify and expand on some of the themes that emerged during analysis of the written comments.

### Quantitative methods

#### Exploratory Factor Analyses

Our analyses started by identifying items linked to a published construct or potentially linked to the construct based on its face validity. The process of organizing the items led to the creation of 3 major meta-categories of neighbourhood items: 1. Physicality; 2. Social; and 3. Resources. Built environment items were used only for descriptive purposes.

A large proportion of items in the observational tool were on a binary scale (Yes/No) and many ordinal-scaled items presented skewed distributions suggesting two or at most three possible values. Therefore, for the purposes of factor analyses, these items were dichotomized. We created composite indicators to include items with very low prevalence (<5% or 9 of 176 block faces). Items that were too rare or could not be included in composite indicators were excluded from the analyses. Items that could vary over the day were observed twice. These were called 'variable items'. We consolidated information of variable items observed on two occasions by indicating presence (or the attribute) observed either in the first or second survey. In each case of an ordinal item, we selected the highest category observed (e.g. '*some *garbage ' was selected over '*little *garbage ').

We conducted exploratory factor analysis to elicit underlying dimensions of the 3 meta-categories in the studied neighbourhoods. Because items were binary, we estimated the tetrachoric correlations and performed dichotomous factor analysis using the weighted least-squares with mean and variance adjustment estimator [32,33]. In brief, tetrachoric correlations assume a threshold model for the observed binary items and latent bivariate normal distribution for each pair of binary items. For example, presence of 'some/a lot of garbage' (observed) is indicated only if the amount of garbage (latent) is greater than a certain threshold (assumed to be consistently evaluated by all raters because of standardized training). During the exploratory factor analysis, items were further excluded because of: (1) high correlation with another item; (2) failure to load in any factor; (3) factor loading <0.40; and (4) loading in multiple factors. The adequacy of the number of factors was evaluated using the chi-square test for overall model fit (p-value > 0.05) and the root mean square residual (RMSR < 0.05). Binary items were compared across neighbourhoods using Fisher's exact tests; analysis of variance and the Wilcoxon-rank sum test assessed differences with respect to factor scores.

Factor analyses were performed using Mplus version 3.1 (Muthen & Muthen, Los Angeles, CA) software; all other analyses were performed using SAS version 9.1 (SAS Institute Inc., Cary, NC).

## Results

### Qualitative Findings

Of the 176 BFs evaluated, raters documented comments corresponding to 112 (64%) of the systematic social observations. The qualitative analyses revealed that raters' comments related to three broad levels of interpretation. These comments described: (1) features of the BF being observed, (2) features of the BF relative to the surrounding neighbourhood, and (3) features of the rater's experience while observing the BF. The qualitative subcommittee revisited the 21 emergent codes and the raw data to determine if it was more useful to fit the themes to the meta-categories derived from the quantitative data. However, all analysts agreed that the qualitative data were much better suited to the three specific levels of interpretation outlined above.

### Features of the BF being observed

Raters expressed concern about misrepresenting a BF by capturing only limited inventory-type data contained within the SSO instrument. As a result, many raters attempted to provide additional contextual information during their observations. An example of a comment which attempted to justify marking a certain response is presented below. In this instance, the rater is uncomfortable with completing Question 47 of the instrument, which documents the presence of 'vacant/undeveloped lots'. The rater wrote,

*"One very big undeveloped lot that looks like it may be used as a park or reclaimed as a park. There's a community vegetable garden within it and a flock of Canadian geese are resting in it and the grass is cut, but I don't see play equipment or any sign denoting that it's a park." *(Eglinton East).

It would have been inappropriate to indicate that the area was a park, yet the response category of 'vacant/undeveloped lots' did not seem to capture the land use adequately in this instance. In previous studies using similar SSO instruments, vacant lots were often presumed to be indicators of physical disorder [7]. In this case, however -- given the evidence of a community garden -- the comment suggests that this land use cannot be easily categorized as either vacant or undeveloped as suggested by the instrument. The community garden not only implies a certain level of physical order in this undeveloped lot, but also hints at a level of social cohesion by the residents' use of common lands. Moreover, this example represents numerous instances within the qualitative data that speak to the challenges and difficulties of capturing neighbourhood characteristics according to the mutually exclusive categories dictated by the SSO instrument. At the same time, the additional contextual information provided by the qualitative data demonstrates the additional strength and depth provided by the use of mixed-methods.

### Features of the BF relative to the surrounding neighbourhood

As discussed previously, a random sample of BFs was examined within each neighbourhood, and raters often expressed in their comments how the BF under observation did or did not appear to correspond with the surrounding area. A couple of examples of such data follow:

*"Very messy block face compared to surrounding neighbourhood" *(South Parkdale)

*"Sense of neighbourhood pride, heritage sign outside on 2 houses and child made artwork outside another with label 'beautiful Weston' " *(Weston)

These comments provide important contextual information, situating the BF observed within the surrounding neighbourhood. Raters' comments indicated that BFs within all neighbourhoods evaluated were characterized by considerable heterogeneity (with the sole exception of Banbury-Don Mills). Furthermore, the comments suggest that this variability or heterogeneity could be seen as positive or negative depending on the particular BF characteristic or feature under observation. This finding was reinforced during the group discussion as raters unanimously agreed that diversity of land use within BFs was viewed as an attribute of an appealing BF. Moreover, raters viewed heterogeneity (in the context of diversity) as a positive attribute of *both *BFs and neighbourhoods. This was especially the case with respect to aesthetic and natural aspects of the physical environment, diversity of architectural styles, and multicultural resident profiles. These findings were somewhat surprising and stand in contrast with previous literature in the area which has emphasized the significant correlation between uniformity and lack of disorder [15].

### Features of the rater's experience while observing the BF

Raters spent hours at a time performing SSOs, and this seemed to provoke reflections about their experiences during data collection. Not surprisingly, the findings stemming from these personal experiences emerged as an independent theme. A characteristic example follows:

*"I talked to two people on the block. A lady saw me looking at the neighbour's driveway and wanted to know what I was doing. She told me that there were lots of historic homes in the area, that this area was really nice. Her neighbour (a soldier in the army) ... started talking about how there are too many taxes, the street's aren't kept up, that Weston Road's a bad neighbourhood - apparently there's a really nice house near it that's not getting sold because there was a shooting 30 ft. away. He then went around and cleaned up not only his litter but the litter at his neighbour's house." *(Weston)

Though seemingly simple in its narrative quality, this example represents a highly complex account of a neighbourhood interaction. Firstly, the neighbours feel comfortable approaching a stranger and the woman appears to exercise vigilance with respect to her neighbour's property. Secondly, the female resident provides a positive account of her neighbourhood, which is then contrasted by the account of the second neighbour, who readily joins the conversation. In addition, there is the stark contrast between the pleasantness of the houses in the area coupled with the story of violence at one nearby residence, which again reinforces the notion of considerable heterogeneity over small spaces and the concept of covert disorder. Finally, the male neighbour takes care of his own yard as well as his neighbour's suggesting social cohesion along the BF.

This example speaks to instances of commonality as well as differences in perception. Such commentary from the qualitative component of the SSO and the retrospective group discussion with raters raised issues relating to their preconceived notions of the neighbourhoods under study - perceptions which were often based on neighbourhood reputation, media accounts and previous personal experiences. Our findings revealed that a rater's preconceived notions about a given neighbourhood could be - and often were - challenged by their fieldwork experiences. In addition, these experiences provided richer contextual information not immediately accessible from straightforward application of the SSO tool, especially in relation to heterogeneity and covert disorder. For example, in the group discussion, one rater commented,

"diversity played definitely, I think we'd all agree, on providing a more positive experience......"

The group discussion revealed that raters felt diversity, positive social interactions, familiarity, aesthetic qualities, structural attributes (e.g. the presence of sidewalks) and a sense of personal safety - all were attributes that contributed to the impression of an appealing BF.

These qualitative findings are crucial to understanding and interpreting the quantitative data, its limitations and strengths, and the performance of the tool in the field. It is to this analysis which we now turn.

### Quantitative Findings

The quantitative analysis assesses the 'performance' of the SSO tool in the field. Individual items were evaluated with respect to their construction and sensitivity, and to determine whether it could be used to distinguish between neighbourhoods, and specifically between neighbourhoods of differing median income. Finally, factor analysis was conducted to determine whether the instrument items could be grouped into meaningful higher-level constructs related to physical and social disorder.

### Descriptive Statistics

The prevalence of the remaining 58 items was calculated and compared 1) between low and middle/high income neighborhoods; and 2) between individual neighborhoods (Table 1). Statistically significant differences (based on income and neighbourhood) were identified for: items regarding the physical condition of the neighbourhood (garbage; cigarette butts; poor condition of buildings, grounds, and public spaces; flow of traffic; number of trees), social aspects of the neighbourhood (the presence of drinking establishments; the presence of drunken, disorderly adults or gangs; residents socializing in mixed racial groups; the presence of languages other than English on the BF), and other factors such as the presence of usable public phones, signs denoting a neighbourhood name, and the presence of toys in private residential grounds. Several items did not demonstrate significant differences between low and high income neighbourhoods, but achieved statistical significance upon stratification by individual neighbourhood. These variables included resident reaction to raters, presence of public courtesies, graffiti, presence of children/teenagers/adults, and others.

**Table 1:** Prevalence of items in 176 block faces by neighbourhood

			Neighbourhood					
	**Total**		**Low Income**				**Middle/High Income**	

**Items (divided by meta-category)**	**%**	**No**.	**EE, N = 39** **%**	**SP, N = 24** **%**	**SJT, N = 9** **%**	**W, N = 43** **%**	**NR, N = 11** **%**	**BDM, N = 50** **%**

***Physicality Items***								

Any Abandoned/Beat-Up Vehicles	8.0	14	0	4.2	0	16.3	9.1	10.0

Some or a lot Garbage Or Odors*^,¶^	43.2	76	43.6	79.2	44.4	48.8	63.6	14.0

Any beer containers or liquor bottles on the street or sidewalks, in yards, or vacant lots	8.5	15	12.8	8.3	11.1	11.6	9.1	2.0

Some/A Lot cigarettes/cigar butts or discarded cigarette packages on any of the streets or sidewalks, in yards/lots or gutters**^,¶^	59.7	105	66.7	91.7	100.0	51.2	63.6	38.0

Any graffiti on any of the buildings, sidewalks, walls, or signs^,¶^	41.5	73	33.3	66.7	100.0	32.6	45.5	32.0

Any Vacant/Undeveloped Land/Lots	11.4	20	12.8	12.5	22.2	4.7	9.1	14.0

Poor/fair/deteriorated condition of the public spaces**^,¶^	63.6	112	69.2	83.3	88.9	79.1	54.6	34.0

Poor/Fair/Abandoned condition of residential buildings**^,¶^	19.3	34	20.5	45.8	55.6	16.3	18.2	2.0

Poor/Deteriorated/Fair overall condition of residential grounds**^,¶^	22.2	39	23.1	45.8	33.3	27.9	18.2	4.0

Poor/Fair overall condition of the vacant/undeveloped property	8.5	15	12.8	12.5	22.2	4.7	0.0	6.0

Poor/Fair condition of commercial buildings*	16.5	29	18.0	25.0	33.3	20.9	9.1	6.0

Any residences with "for sale" or "for rent" signs*	27.8	49	33.3	37.5	22.2	34.9	36.4	12.0

Any commercial/industrial buildings with "for sale" or "for rent" signs*	10.2	18	5.1	25.0	11.1	11.6	9.1	6.0

								

***Social Items***								

Presence of a police officer or private security guard	9.7	17	12.8	16.7	33.3	4.7	9.1	4.0

Presence of adults loitering or selling drugs	8.0	14	10.3	8.3	33.3	7.0	9.1	2.0

Presence of gangs or drinking, drunken or disorderly groups of adults**^,¶^	6.3	11	7.7	8.3	55.6	2.3	0.0	0.0

Presence of indications of neighbourhood uniformity	10.2	18	2.6	12.5	22.2	7.0	0.0	0.0

Presence of signs which denote a neighbourhood name*^,¶^	10.2	18	5.1	37.5	33.3	4.7	9.	2.0

Presence of neighbourhood crime watch signs	19.3	34	20.5	8.3	33.3	16.3	27.3	22.0

Presence of no trespassing or security warnings signs	38.1	67	30.8	45.8	44.4	39.5	45.5	36.0

Presence of any residences with signs indicating they are protected by dogs*	14.2	25	23.1	16.7	11.1	18.6	0.0	0.0

Presence of any residences with some form of decoration (e.g. decorated gardens/balconies, nameplates, window boxes)?	76.7	135	82.1	66.7	66.7	76.7	81.8	78.0

Presence of any residential building with a fence or border	74.4	131	84.6	62.5	66.7	76.7	81.8	70.0

Presence of any residential buildings with window bars or door grates^¶^	17.6	31	18.0	33.3	33.3	9.3	63.6	4.0

Presence of any residential buildings with signs indicating they are protected by private security services	51.1	90	56.4	50.0	44.4	51.2	18.2	56.0

Presence of any commercial/industrial buildings with barred windows	10.2	18	12.8	20.8	22.2	7.0	9.1	4.0

Resident react to the presence of raters^||^	39.2	69	56.4	58.3	33.3	25.6	36.4	30.0

Three or more people present on the block face	35.8	63	20.5	37.5	100.0	37.2	63.6	28.0

Heard or saw a language other than English on the block face*^,¶^	30.1	53	35.9	41.7	77.8	25.6	36.4	14.0

Presence of any people socializing in mixed racial groups**^,||^	29.6	52	25.6	45.8	66.7	34.9	18.2	16.0

Presence of adults walking, socializing, doing home repair, sitting on the porch, supervising children, exercising, or patronizing businesses on the block face^¶^	83.5	147	76.9	95.8	100.0	81.4	100.0	78.0

Presence of signs advertising cultural, political or social events or neighbourhood helper signs^¶^	15.9	28	5.1	41.7	66.7	9.3	45.5	2.0

Presence of children^¶^	49.4	87	51.3	37.5	88.9	53.5	90.9	34.0

Presence of teenagers^¶^	37.5	66	30.8	25.0	88.9	53.5	63.6	20.0

Presence of public seating	10.8	19	7.7	16.7	22.2	7.0	18.2	10.0

Presence of children or teenagers in the parks or playgrounds^||^	9.7	17	7.7	0.0	44.4	14.0	9.1	6.0

Presence of children or teenagers in playing in public spaces other than parks or playgrounds^¶^	13.1	23	7.7	4.2	33.3	9.3	63.6	10.0

Presence of any residential grounds with toys or play equipment*^,¶^	25.0	44	33.3	0.0	0.0	20.9	36.4	36.0

								

***Resources***								

Access to public transportation on block face*	26.1	46	23.1	37.5	44.4	32.6	9.1	18.0

Presence of public courtesies, such as public seating, trash cans, newspaper dispensers and public pay phones^¶^	33.5	59	23.1	58.3	77.8	30.2	36.4	24.0

Presence of usable public phones**^,¶^	10.8	19	12.8	16.7	44.4	11.6	0.0	2.0

Presences of handicap accessibility facilities such as sidewalk ramps, wheel-trans stops, or reserved parking	18.2	32	10.3	12.5	55.6	20.9	18.2	18.0

Presence of any bicycle facilities such as bike lanes and parking stands^¶^	12.5	22	2.6	33.3	44.4	9.3	27.3	4.0

Presence of a park or playground.	14.2	25	12.8	4.2	44.4	16.3	9.1	14.0

Presence of other play spaces (sports fields or children playing in other public spaces)^¶^	30.1	53	18.0	4.2	55.6	41.9	72.7	28.0

Good or well-kept public spaces**^,¶^	31.8	56	20.5	12.5	11.1	20.9	45.5	60.0

Playgrounds/parks in good or excellent condition	8.0	14	7.7	0.0	0.0	9.3	9.1	12.0

Other play spaces in good or excellent condition*^,¶^	19.9	35	15.4	0.0	11.1	23.3	54.6	24.0

Presence of commercial establishments^||^	26.7	47	23.1	50.0	44.4	25.6	27.3	16.0

Presence of institutions (eg. Schools, churches, medical clinics, libraries)	19.9	35	18.0	29.2	33.3	23.3	27.3	10.0

Presence of drinking establishments*^,¶^	10.2	18	12.8	33.3	11.1	4.7	18.2	0.0

Light or very light traffic**^,||^	63.1	111	59.0	41.7	44.4	62.8	72.7	78.0

								

***Built Environment***								

Presence of single family homes^||^	66.5	117	76.9	50.0	22.2	74.4	81.8	64.0

Presence of buildings greater than two stories tall^¶^	30.7	54	7.7	70.8	100.0	18.6	90.9	14.0

Greater or equal to 20 residences on the block face^¶^	54.0	95	61.5	16.7	33.3	37.2	27.3	62.0

Greater or equal to 20 trees on the block face*^,¶^	31.3	55	38.5	45.8	33.3	41.9	18.2	12.0

All residences have access to a private yard or garden^¶^	54.0	95	79.5	8.3	0.0	53.5	27.3	72.0

Greater or equal to 50% of residences have a private balcony, porch or terrace^¶^	47.2	83	25.6	58.3	77.8	41.9	36.4	60.0

EE = Eglington East, SP = South Parkdale, SJT = St. Jamestown, W = Weston, NR = North Riverdale, BDM = Banbury Don Mills

* Significant difference p < .05, two-sided Fisher's exact test comparing low income and high income neighbourhoods

** Significant difference p < .01, two-sided Fisher's exact test comparing low income and high income neighbourhoods

^|| ^Significant difference p < 0.05, Fisher's exact test comparing all six neighbourhoods

^¶ ^Significant difference p < 0.01, Fisher's exact test comparing all six neighbourhoods

### Factor Analysis

#### Physicality Meta Category

We obtained 4 competing, interpretable, one-factor models (Table 2). Each model was evaluated on the basis of theoretical justification and relative fit indices (RMSR) (3 yielded RMSR slightly above 0.05 and one had an RMSR < 0.05). The models had comparable RMSR values, but Models 1, 2, and 3 omitted items that were important in discriminating between neighborhoods on the basis of physical features, namely the amount of cigarette butts and the condition of abandoned buildings (see prevalence Table 1). Model 4 included these items and had similar factor loadings for the common items. Therefore we considered Model 4 with 7 items as the most interpretable model, capturing the greatest number of physical BF characteristics, with no loss of relative fit to the data. The factor outlined in Model 4 was named "Physical Decay and Disorder" and retained for use in neighborhood comparisons.

**Table 2:** Exploratory factor analysis of Physicality items

**Item**	**Model 1**	**Model 2**	**Model 3**	**Model 4**
			
Poor/Fair condition of commercial buildings	0.725	0.711	0.766	0.749
Poor/fair/deteriorated condition of public spaces	0.562	0.604	0.597	0.618
Poor/fair/abandoned condition of residential buildings		0.640		0.630
Some or a lot of Garbage or Odors	0.923	0.907	0.814	0.826
Any beer containers or liquor bottles	0.660	0.644	0.696	0.694
Any graffiti	0.496	0.505	0.547	0.547
Some/A Lot cigarettes/cigar butts			0.937	0.930
				
χ^2 ^p-value	0.949	0.938	0.870	0.913
RMSR	0.043	0.054	0.053	0.060

#### Social Meta Category

It was not possible to extract a model with interpretable factor structure, overall or stratified by income level. Factors reported in published studies [7,21] were not validated in this sample.

#### Resource Meta Category

Fifteen Resource items could not be factor analyzed in the same model. Two subsets of items yielded 2 separate one-factor models (Table 3). Items that reflected accessibility within the neighbourhood (bike facilities, and public transport) and social activities (cultural events and establishments where alcohol was served) constituted the "Neighbourhood Social Accessibility" factor.

**Table 3:** Two one-factor models of separate subsets of Resource items

*One-Factor Model*	*Factor loading*	*χ^2 ^p-value*	*RMSR*
***"Neighbourhood social accessibility"***			
Access to public transportation on the block face	0.731	0.523	0.0547
Presence of any bicycle facilities such as bike lanes and parking stands	0.893		
Presence of signs advertising cultural, political or social events or neighbourhood helper signs	0.531		
Presence of drinking establishments	0.818		
			
***"Recreational opportunities"***			
Good or well-kept public spaces	0.795	0.5644	0.0516
Parks/playgrounds in good or excellent condition	0.609		
Other play spaces in good or excellent condition	0.589		
Light or very light traffic	0.648		

The factor labeled "Recreational opportunities" was composed of *Public spaces condition, Good parks condition, Good other play space condition *and *Light/Very Light Flow of Traffic *items. This factor reflected the opportunities for recreation and safe play that were afforded by the block face.

### Comparing Neighbourhood Physical Decay and Disorder

**Figure 2:**
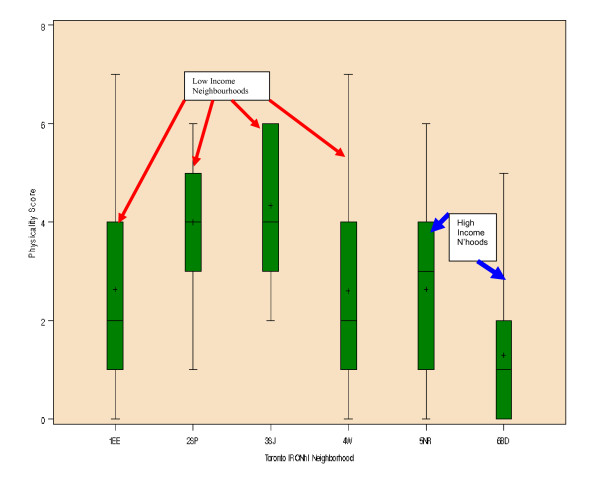
**Box and whisker plot comparing Physical Decay and Disorder Scale by neighbourhood**. Median, IQR and range of Physical Decay and Disorder Scores stratified by neighbourhood. Physical Decay and Disorder score is the sum of 7 items: Poor/Fair condition of commercial buildings; Poor/fair/deteriorated condition of public spaces; Poor/fair/abandoned condition of residential buildings; Some or a lot of Garbage or Odors; Any Graffiti; Any beer containers or liquor bottles; Some or a lot of cigarette butts.

A scale was created from the Physical Decay and Disorder factor identified in the Physicality exploratory factor analysis. A summary score for each BF was created by adding one point for each of the seven items endorsed. The potential minimum score was 0 and the potential maximum score was 7. A box and whisker plot was generated comparing Physical Decay and Disorder scores for BFs stratified by neighbourhood (Fig. 2).

Mean Physicality scores across neighbourhoods were statistically different (ANOVA, p < 0.001). As variances among neighbourhoods were equal (Levene test p-value = 0.193), we compared pairs of neighbourhoods using Tukey's Honestly Significant Test at α = 0.05. Banbury-Don Mills had significantly lower average scores than Eglinton East, South Parkdale, St Jamestown and Weston; Eglinton East had lower average scores than South Parkdale; and South Parkdale had lower average scores than Weston. The Wilcoxon rank-sum test using a Bonferroni correction for multiple comparisons yielded the same results. Mean Social and Resource scores could not be compared across neighbourhoods because it was not possible to extract models with interpretable factor structures for either of these meta-categories.

## Discussion

This study sought to evaluate the performance of an SSO instrument in the context of six Toronto neighbourhoods. This instrument was based on scales and constructs from prior studies largely generated in US settings. The factor analysis based on the quantitative data, taken together with the qualitative findings, raises important questions and concerns with respect to the 'transferability' of such constructs to the Toronto context.

### Interpreting the Data

The qualitative component of this study contributes to the literature on SSO by acknowledging the inherently subjective nature of neighbourhood observations (and recognizing the positive contributions of such subjective data sources) - a consideration absent from the literature to date. The inclusion of qualitative methods to our study demonstrates the depth and richness of analysis that can be obtained by using a mixed-methods approach for SSOs. To our knowledge this is one of the first studies to incorporate qualitative observational findings and researcher reflections into an investigation employing quantitative systematic observational tools. As such it represents an important contribution to our understanding of neighbourhood evaluation. One British study (Morrow, 2000) [34] has examined youth residents' self-reported perceptions of neighbourhood physical context and its impact on youth well-being using qualitative methods, however we use these techniques to tap raters' perceptions, not those of residents. Furthermore, Morrow does not attempt to tie her findings to observational data collected in the neighbourhoods under study[34].

Analysis of the qualitative data revealed three broad themes related to use of the SSO instrument in the field: (1) features of the BF being observed, (2) features of the BF relative to the neighbourhood, and (3) features of the rater's experience while observing the BF. Each of these themes poses further questions with regard to the utility of the SSO instrument in the Toronto context and challenges some of the assumptions upon which SSO research has been based to date. In particular, our qualitative findings urge us to question the meaning of the quantitative results with respect to the underlying social processes relating to neighbourhood disorder.

The first theme (*features of the BF being observed*) problematizes the 'objective' nature of this form of data collection. There were numerous instances where raters found it challenging to appropriately characterize BFs, and they feared misrepresenting BFs - and the implications of such misrepresentations when drawing comparisons between neighbourhoods. This may be a concern when examining urban settings which have low levels of severe disorder and substantial intra-neighbourhood variation as was noted in our study. The quantitative findings are more readily interpretable in light of the complex nature of the data collection process. For example, the factor 'physical decay and disorder' was the only one which resembled the constructs generated in the US studies. The high prevalence of cigarette butts (noted by raters in *all *neighbourhoods) accounted for a significant proportion of this factor. The physical decay/disorder construct was further influenced by the high prevalence of ratings of 'poor/fair/deteriorated condition of public spaces' (with this rating assigned to 63.6% of BFs). The standardized instructions may have skewed these results in favor of a preponderance of 'fair' ratings. The standardized instructions indicated that any street, sidewalk, public transit stop, public parks or grounds, public schools or any non-private land should be marked in 'fair' condition if it showed irregular maintenance (including those with even small amounts of cracked concrete or paint or moderately overgrown vegetation) and overall the space was "in decent condition, but (rater) would recommend additional upkeep." Such instructions logically resulted in most raters ranking public spaces as being in fair condition. However it is questionable whether this degree of disorder on its own would result in a negative experience for persons using the BF. It was discomfort with this type of rating that raters' comments reflected.

The second theme addressing *features of the BF relative to the surrounding neighbourhood *challenges commonly held assumptions regarding neighbourhood homogeneity as these relate to Toronto neighbourhoods. The qualitative findings underline the importance of heterogeneity within the neighbourhood and how this sense of heterogeneity may impact the overall impression of a specific area within a neighbourhood or the entire neighbourhood itself. The impact of such heterogeneity challenges the classic understanding of a neighbourhood which presupposes certain levels of homogeneity within a specified bounded area [35]. It may also provoke questions concerning the significance of the BF or smaller bounded communities within neighbourhoods in the presence of considerable neighbourhood variability. Certainly the quantitative findings reflected neighbourhood heterogeneity as well. The creation of the 'physical decay and disorder' scale revealed that low-income neighbourhoods were more likely to be characterized by greater levels of disorder/decay than middle/high-income neighbourhoods. However, the box-and-whisker plots indicate that considerable heterogeneity exists *within *each neighbourhood, regardless of income. Taken together, these mixed-method findings suggest that the impact of concentrated disorder (evident in smaller pockets within neighbourhoods) may be diluted when describing neighbourhoods more broadly, implying a notion of "covert disorder" in the Toronto setting.

The third theme examining *features of the rater's experience while observing the BF *speaks to the access to information that would not have been otherwise obtained by using a purely quantitative approach or even the use of other data collection methods such as observations performed by driving through neighbourhoods [7,21]. The fact that raters performed observations on foot, walking up and down a BF numerous times, offered residents the opportunity to interact with them, in turn yielding detailed narrative accounts. When raters did not engage with residents, their very presence on the BF provided them access to observations that might have escaped notice using other methods of data collection such as drive-by observation. This is because, as observers on foot, raters could observe in 360° over a longer period of time, since most observations required at least 30 minutes for completion.

Moreover, the narrative accounts of raters' experiences often revealed unanticipated information concerning the neighbourhoods. For example, raters suggested in group discussion that evidence of extreme social disorder was often fleeting to the outsider--erupting to the surface at intervals, but not always obvious at first glance. As well, information obtained through interaction and observation frequently challenged raters' preconceived notions of the BF or neighbourhood. The qualitative findings reinforce the importance of heterogeneity and covert disorder in explaining features of the neighbourhoods under study. By capturing the raters' experiences in a systematic way, the qualitative portion of our study was able to access yet another level of rich contextual information that supported and helped in interpreting our quantitative results.

The quantitative findings also provided new insights regarding SSO. The generation of the two distinct constructs from the resources meta-category is an original contribution to our understanding of neighbourhoods. The 'neighbourhood social accessibility' factor speaks to neighbourhoods as dynamic entities rather than static ones. It reflects the ease with which one can enter and leave a neighbourhood and - coupled with signs advertising social and cultural events as well as the presence of drinking establishments - suggests features of neighbourhoods that make them desirable places for both residents and non-residents alike. The 'recreational opportunities' factor represents a related construct in that places to play or meet in public spaces without being overwhelmed by traffic (and its attendant congestion, parking difficulties, noise and pollution) might also prove appealing to residents. In a forthcoming study employing concept mapping (Sheppard et al: "Are Canadians influenced by their urban neighbourhoods? Neighbourhood characteristics and their perceived impact on self-rated mental well-being," submitted)- which asked residents for their perspectives on neighbourhoods and mental health - residents indicated that pedestrian-friendly neighbourhoods, accessible by public transit or other means, with plenty of public services, places to meet and occasions to celebrate - all were reported as contributing to residents' mental well-being. The quantitative findings in the present study suggest that such neighbourhood factors are 'observable' (physically quantifiable) and are important to residents.

Within the quantitative analysis, we were able to identify some differences between low and high income neighbourhoods on the factor 'physical decay and disorder' and on some individual items. Several items did not demonstrate significant differences between low and high income neighborhoods, but achieved statistical significance upon stratification by individual neighbourhood. These variables (including resident reaction to raters, presence of public courtesies, graffiti, signs advertising cultural or social events, the presence of children, teenagers and adults, features of the built environment) may be features of neighbourhoods that are not necessarily linked to income, but are rather descriptors of the unique character of neighbourhoods. Conversely, several items were only statistically significant upon stratification by income (poor/fair condition of commercial buildings, buildings for sale or rent, residences protected by dogs). These items may be more strongly linked to income than neighbourhood, or there may be insufficient power to resolve them.

It was not possible to extract a model with interpretable factor structure for the Social meta-category. Possible explanations include lack of power due to rare items or small sample size, or an artifact due to how the items were dichotomized. However this may also reflect fundamental differences between the neighbourhoods included in the present investigation and the neighbourhoods that were used in the Chicago and Baltimore studies - meaning that the constructs of Territoriality and Social Disorder may not be applicable to Toronto.

By combining the quantitative and qualitative analyses, a number of interesting points for discussion are posed. With respect to the transportability of previously employed SSO tools into a Canadian context, it is fair to ask whether the notion of 'social disorder' is the most appropriate to this setting [36]. In particular, the choice of variables included in the tool and how these were operationalized were of concern to raters. For example, raters were concerned by the limited ability of the SSO instrument to capture certain characteristics of the BF that they felt were important for the Toronto setting, such as the aesthetic appeal of a BF (e.g. the degree of order and diversity of land use), the rater's sense of personal safety, and their experiences during data collection. In contrast, as elicited during the group discussion, many raters felt that items within the SSO tool relating to extreme physical or social disorder were not as relevant to the study of Toronto neighbourhoods, but that these items accounted for a considerable proportion of the overall observation. Oreopoulos (2005) also posited differences in neighbourhood disorder as experienced by Canadian and US residents [23]. Instead of focusing on disorder, the mixed method findings in our study suggest that perhaps the notion of 'order' may be more pertinent in the context of Toronto neighbourhoods (but not 'order' conceived of as a mere corollary to that of 'disorder' premised in most of the SSO literature). We caution that the raters' reflections are those of a very select group of participants - academic researchers, not laymen. Nevertheless, the positive emphasis that all raters gave to diversity (as an appealing attribute at both the BF and neighbourhood level) challenges prevailing planning notions that stress uniformity. The qualitative data therefore help to highlight and reinforce specific challenges regarding the transfer and subsequent application of an SSO tool from one urban context to another. In the case of Toronto, additional variables corresponding to more specific concepts of safety, aesthetic appeal and order, and heterogeneity might be considered in any future revision of the SSO instrument.

Anecdotally, it is not unusual for visitors to Toronto (or even agency representatives providing funding to low-income inner-city neighbourhoods) to ask "when are we going to get to the 'bad' neighbourhood?" As such, relative disorder (both physical and social) and decay are not always obvious to the casual observer. This is not to suggest that Toronto does not have its share of both physical and social disorder. For example, while few homeless people were observed during the study, we know that Toronto has many homeless residents [37]. Rather the tool failed to capture this facet of city life. If we are to use the concept of disorder (rather than order), perhaps a recognition of the *concealed *nature of disorder (particularly social disorder) is more reflective of the Toronto experience.

Taken together, our qualitative and quantitative findings compel us to interrogate the theoretical assumptions underlying social and physical disorder in a Toronto context. As such, we propose that alternative theoretical concepts might be more relevant to this setting given the complexity of the phenomena under investigation. For example, the finding of considerable heterogeneity and and the discussions amongst raters concerning 'covert disorder' *might *suggest that residents perceive their local BF or immediate surroundings as more representative of their functional neighbourhood. From this perspective, the concept of smaller functional geospatial communities bounded within traditionally defined neighbourhoods may very well have a substantial impact on any conceptual framework attempting to describe how neighbourhoods affect health - particularly as they relate to health in Toronto or similar urban settings.

This observational study (like others before it) chose to focus on the facades of BFs as units for observation. Given the questions raised here regarding the nature of disorder, it is fair to ask whether we are looking for disorder in the 'right' places. Perhaps we should be sampling the considerable network of 'back alleys' that are a staple of Toronto's inner-city neighbourhoods (a suggestion offered by raters during the group discussion). The data from our forthcoming concept mapping study (Sheppard et al.: "Are Canadians influenced by their urban neighbourhoods? Neighbourhood characteristics and their perceived impact on self-rated mental well-being," submitted) suggests that residents of apartment buildings (particularly high-rise buildings) include the internal spaces between apartments (lobbies, elevators, common areas) as important features of 'neighbourhood' for them. Perhaps we need to adapt a tool that will capture both 'internal' and 'external' neighbourhood characteristics (and the relative order or disorder therein). What is the 'appropriate' level of observation when evaluating neighbourhoods?

As noted already, this study has a number of important limitations. We were unable to generate factors for the social meta-category, with potential explanations including: a lack of power (secondary to either low prevalence or small sample size), artifact from dichotomizing the variables, or a fundamental problem with the construct of social disorder underlying the adapted scales (adapted from US and UK contexts).

Another important question that we cannot answer by this investigation relates to linkages between the constructs generated by SSO and health. This study rather represents an initial step in understanding the utility and applicability of these tools in different contexts. As such our findings will assist health researchers in interpreting the findings they acquire when using these measures. It is important to note that the nature of the relationship between neighbourhoods and health has been difficult to delineate and such linkages are no doubt complex. Given the increasing interest in this area of research and the use of SSO tools, improved understanding of the instruments themselves (evaluating both their strengths and limitations) is a valuable contribution. For example, if we take the prevalence of homelessness and relate it to health, for example, what are the attributes at the individual level that contribute to vulnerability, and what aspects of place make individuals vulnerable? These are questions which will only be answered by studies employing a variety of methodologies (observation, concept mapping, surveys, interviews, document analysis, policy analysis, etc.), such as Klinenberg's approach of 'social autopsy' [38].

Our employment of mixed quantitative and qualitative methods represents a unique contribution to the field of neighbourhoods-and-health research. The qualitative findings enhance our understanding of the quantitative data and analyses, but also add new and important information regarding raters' experiences in conducting such research. Both forms of data (and their interpretation) contribute to our understanding of neighbourhood-level characteristics and both pose important questions regarding the best ways to characterize neighbourhoods.

## Conclusions

This study tested the performance of an SSO tool (adapted from previous studies in the US and UK) in a Canadian context. Based on our analysis, the SSO tool as implemented here demonstrated considerable shortcomings when applied to these six Toronto neighbourhoods. While we were able to generate three 'factors' using the quantitative data (physical decay and disorder, neighbourhood social accessibility, and recreational opportunities), only the first reflected the findings of other investigators in that it was able to differentiate between low and higher income neighbourhoods. Unlike investigators in other jurisdictions, we found relatively few instances of items linked to 'disorder' in implementing the SSO tool. Coupled with the qualitative findings, which related to raters' experiences of conducting neighbourhood observations, our study raises important questions regarding the theoretical premises (such as social disorder) underlying much neighbourhoods-and-health research. To our knowledge, this study is the first to employ a qualitative component to SSO. Our findings demonstrate that US and UK-generated constructs are not readily adaptable to a Canadian context, and suggests other constructs that may be more appropriate to apply in this setting. It poses some interesting directions for future research, namely the development of new instruments, different levels of observation, new methodological approaches (particularly greater use of mixed methods), and new theories with which to understand neighbourhood-level effects on health.

## Competing interests

The authors declare that they have no competing interests.

## Authors' contributions

JAP participated in the design of the study (including conceptualization of the qualitative components), data collection and analysis, and drafted the manuscript. GS participated in the data collection and analysis and assisted with preparing the manuscript. ANS carried out data collection and analysis (quantitative and qualitative), and assisted in manuscript preparation. RN performed the statistical analyses and contributed to the study design and manuscript preparation. PB assisted with data collection and analysis and assisted with manuscript preparation. AJ assisted with the qualitative data analysis. QZ contributed to data collection and analyses. AS contributed to data collection and analysis (qualitative and quantitative). JR assisted with data collection and quantitative analyses, as well as manuscript preparation. PO conceived the study, directed its design and coordination, contributed to the analyses and assisted with manuscript preparation. JD (with PO) conceived the study, directed its design and coordination, contributed to the analyses and assisted with manuscript preparation. All authors read and approved the final manuscript.
